# Supplementary figures and images for: Genetic analysis of osteoblast activity identifies Zbtb40 as a regulator of osteoblast activity and bone mass
Source: PLoS Genet. 2020 Jun 4;16(6):e1008805. doi: 10.1371/journal.pgen.1008805 (PMC7326283; doi:10.1371/journal.pgen.1008805)

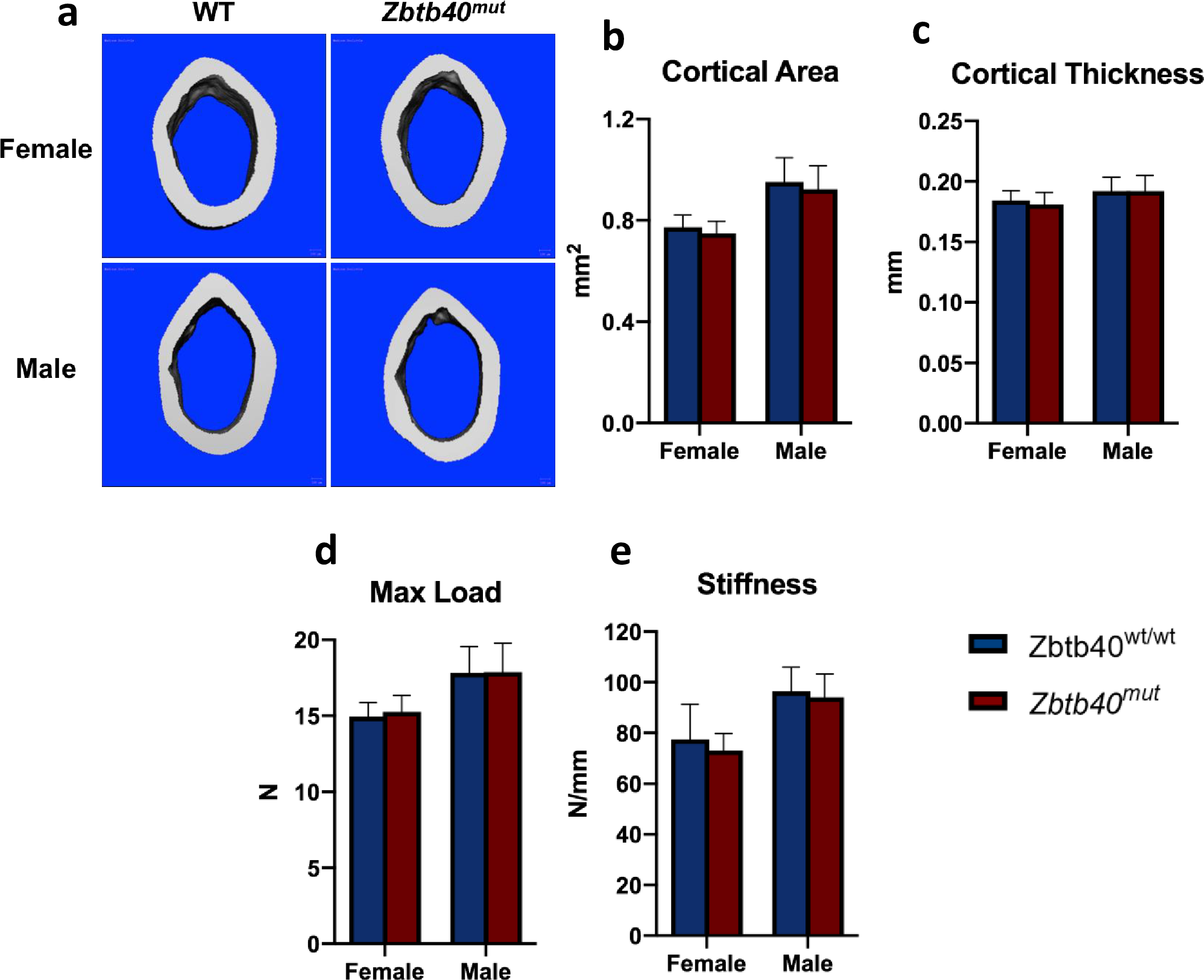

Supplement: S1 Fig — Wnt4fl/fl Prrx1-Cre mice show reductions in femoral trabecular number in both sexes and cortical area in females: (A) Confirmation of Wnt4 deletion by floxed allele recombination in the femur of a Cre positive wt/fl mouse by RT-PCR (B) Whole body and (C) Femoral BMD of female (wt/wt n = 19, fl/fl n = 19) and male (wt/wt n = 5, fl/fl n = 17) mice measured by DXA at 16 weeks of age. (D,E,F) Trabecular number (Tb.N) values and 3D reconstructions of the femoral metaphysis from female (wt/wt n = 9, fl/fl n = 10) and male (wt/wt n = 4, fl/fl n = 9) mice. (G,H) Cortical area (Ct.Ar) calculations and 3D reconstructions of the femoral mid-diaphysis in female mice. * = p<0.05 ** = p<0.01 *** = p<0.001—Unpaired T test. (TIF) [file pgen.1008805.s001.tif]

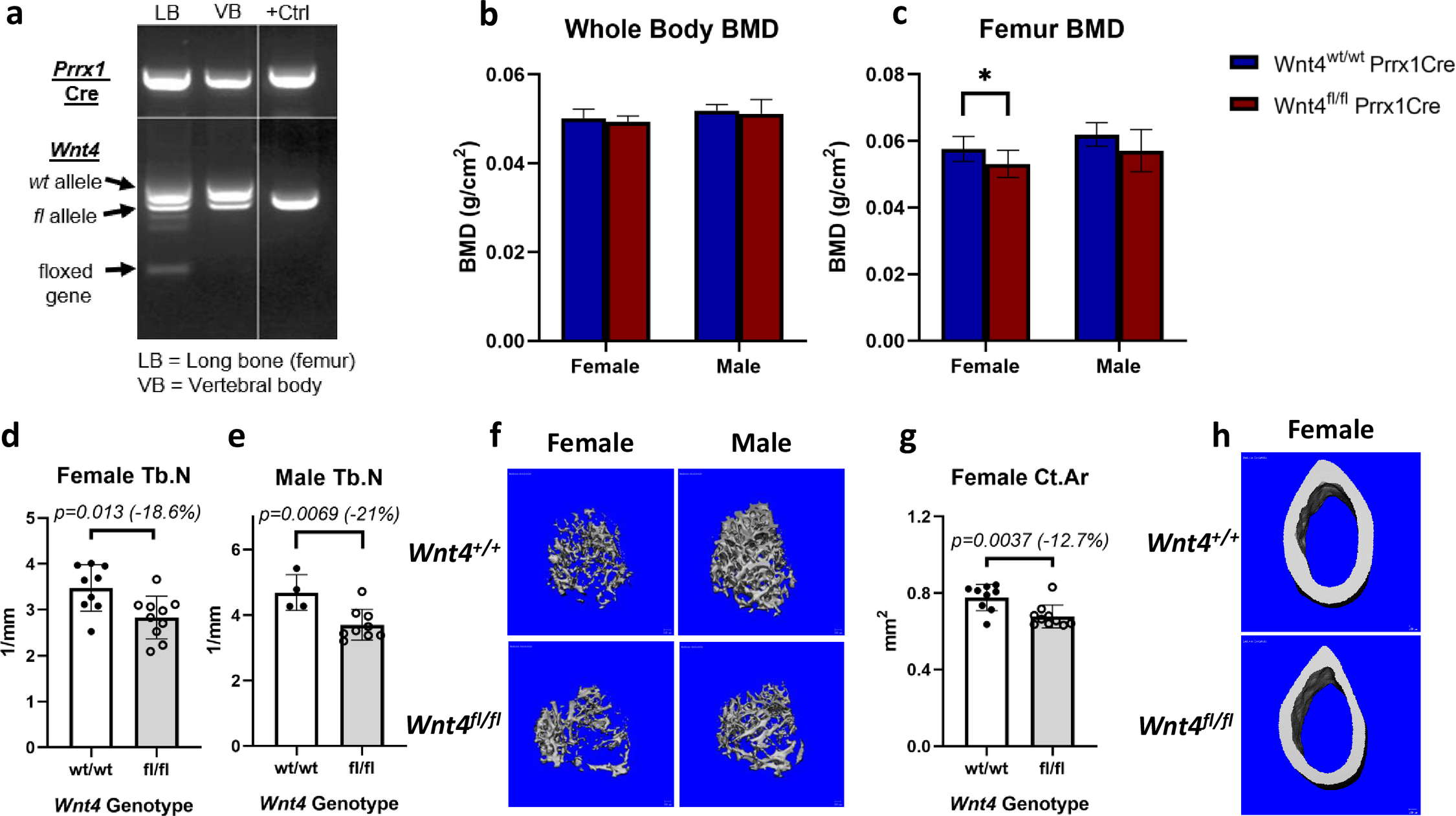

Supplement: S2 Fig — Zbtb40mut mice show no changes in femoral cortical bone mass or strength: (A, B, C) 3D reconstructions of femur mid-diaphyseal cortical bone and calculations for cortical area and cortical thickness (wt/wt n = 9, mut/mut n = 9 per sex, 16 weeks of age). (D, E) Max load and stiffness calculations from biomechanical 3-point bending test (female—wt/wt n = 9, mut/mut n = 6, male—wt/wt n = 8, mut/mut n = 9, 16 weeks of age). (TIF) [file pgen.1008805.s002.tif]
